# Safety of TeaCrine®, a non-habituating, naturally-occurring purine alkaloid over eight weeks of continuous use

**DOI:** 10.1186/s12970-016-0113-3

**Published:** 2016-01-13

**Authors:** Lem Taylor, Petey Mumford, Mike Roberts, Sara Hayward, Jacy Mullins, Stacie Urbina, Colin Wilborn

**Affiliations:** Department of Exercise and Sport Science, Human Performance Lab & Exercise Biochemistry Lab, University of Mary Hardin-Baylor, Belton, TX USA; School of Kinesiology, Auburn University, Auburn, AL USA

**Keywords:** Theacrine, Safety, Habituation

## Abstract

**Background:**

Theacrine (1,3,7,9-tetramethyluric acid) is a purine alkaloid found in certain coffee (Coffea) species, fruits (Cupuacu [Theobroma grandiflorum]), and tea (Camellia assamica, var. kucha) that has anti-inflammatory, analgesic, and neuro-locomotor properties. Recent preliminary research has also reported increased feelings of energy, reduced fatigue, and strong effects on improving focus, concentration, and motivation to exercise. The purpose of this study was to examine the safety and non-habituating effects of TeaCrine®, a nature-identical, chemically equivalent bioactive version of theacrine.

**Methods:**

Sixty healthy men (mean ± SD age, height, weight: 22.9 ± 4.7 years, 183.5 ± 9.2 cm, 86.5 ± 13.7 kg) and women (22.3 ± 4.5 years, 165.2 ± 12.3 cm, 69.0 ± 17.4 kg) were placed into one of three groups: placebo (PLA, *n* = 20), 200 mg TeaCrine® (LD, *n* = 19) or 300 mg Teacrine® (HD, *n* = 21) and ingested their respective supplement once daily for 8 weeks. Primary outcomes were fasting clinical safety markers (heart rate, blood pressure, lipid profiles, hematologic blood counts, biomarkers of liver/kidney/immune function) and energy, focus, concentration, anxiety, motivation to exercise, and POMS measured prior to daily dosing to ascertain potential tachyphylactic responses and habituation effects. Data were analyzed via two-way (group × time) ANOVAs and statistical significance was accepted at *p* < 0.05.

**Results:**

All values for clinical safety markers fell within normal limits and no group × time interactions were noted. No evidence of habituation was noted as baseline values for energy, focus, concentration, anxiety, motivation to exercise, and POMS remained stable in all groups across the 8-week study protocol.

**Conclusions:**

These findings support the clinical safety and non-habituating neuro-energetic effects of TeaCrine® supplementation over 8 weeks of daily use (up to 300 mg/day). Moreover, there was no evidence of a tachyphylactic response that is typical of neuroactive agents such as caffeine and other stimulants.

## Background

TeaCrine® is a nature-identical, chemically equivalent bioactive version of theacrine. Theacrine is a purine alkaloid that is converted from caffeine by hydration, oxidation and methylation [1] and it is thought to influence the central nervous system as a neuroactive ingredient. Theacrine is the primary extract from the cultivated tea plant *Cameilla kucha*, and has recently been studied as an ingredient that may have various therapeutic and medicinal uses. Acute supplementation is theorized to enhance mood state, increase energy production, heighten mental focus, and strengthen motivation. In various rodent models, theacrine has been shown to increase dopamine levels, decrease ROS (reactive oxidative species) and inflammation, decrease adenosine, and modulate other neurotransmitters. However, there is limited research available on chronic theacrine supplementation in humans, with only one poster presentation currently published that addressed effects from acute (single dose) supplementation. Moreover, to our knowledge there is no published research in humans that examines the safety of chronic theacrine supplementation.

The current research on theacrine is isolated in rodent models with the exception of only conference proceedings poster currently published on one acute dose study that aimed to assess the cognitive and psychometric responses in humans. The preliminary abstract data in this trial indicated that acute supplementation of 200 mg of TeaCrine® had beneficial effects on increased energy and reductions in fatigue [2]. Although preliminary, this data along with the various data in animals is interesting and suggests a possible role for the utilization of theacrine as a therapeutic supplement for augmenting cognitive and physical performance [3]. In regards to safety of ingestion, there are currently no clinical trials that have looked at the potential effects, positive or negative, that TeaCrine® supplementation may have on hemodynamics, blood chemistry profiles including liver function, lipid panels, complete blood counts (CBC), etc. in humans. Animal model research has evaluated the toxicity of theacrine and determined it is safe for ingestion [4] and has not been shown to have any negative effects on heart rate and blood pressure in rats [5].

Because theacrine is a caffeine derivative and extracted from tea, its potential effects on body composition are worth exploring. The inclusion of caffeine and tea extracts, primarily green tea in the form of EGCG, is a commonly studied method to influence energy expenditure and body composition. Recent research has shown that ingestion of these types of ingredients in various combinations can increase resting energy expenditure [6], improve body composition [7], enhance exercise performance [8], and improve cholesterol profiles [7] while showing no ill effects on safety parameters [9]. Despite most of this data utilizing multi-ingredient formulas, because of the nature of the compound it is of interest to evaluate if theacrine has any similar effects on body composition profiles.

Collectively, the limited availability of data on theacrine ingestion in human models has led to the research questions being evaluated in this clinical trial, namely to examine the effects of various doses of TeaCrine® on safety (blood chemistry, hemodynamics, ECG functioning), mood profiles, and body composition. Additionally, it is of interest to assess the dosing response on any potential tachyphylactic and/or discontinuation effects that are commonly seen with ingestion of caffeine and other neuroactive stimulants.

## Methods

### Experimental approach to the problem

A randomized, placebo controlled, double-blind study was used to compare the effects of consuming a low dose of Teacrine® (TC-LD), a high dose of Teacrine® (TC-HD) or a placebo (PLA) for 8 weeks on apparently healthy, recreationally active, regular caffeine consuming, males and females. The following dependent variables were assessed to determine supplementation-induced differences based on the three groups: clinical safety markers, heart rate, blood pressure, questionnaires (VAS, Yale PAQ and POMS) and body composition. Subjects were instructed not to change their exercise habits or their standard dietary habits. The design allowed for examination of the effects of Teacrine® in a low and high dose with no diet or exercise regimen manipulations.

### Subjects

Sixty men and women (mean ± SD age, height, weight, body fat percentage: 22.55 ± 4.6 years, 174.09 ± 12.4 cm, 77.47 ± 17.4 kg, 23.4 ± 9.9 %) participated in this study. Subjects were not allowed to participate in this study if they had any metabolic disorder including known; heart disease, arrhythmias, diabetes, cancer; if they were taking medications related to chronic disease; if they were taking or had taken dietary supplements (other than multi-vitamins and/or minerals) within 8 weeks prior to enrollment; if they had participated in another clinical trial within 8 weeks prior to enrollment; if they had any known allergies or sensitivity to any ingredient in the test product; and, if they were currently pregnant, nursing or became pregnant during the duration of the study. Subjects were asked to maintain their normal dietary intake and exercise habits for the duration of the study. Subjects meeting eligibility criteria were informed of the requirements for the study and signed approved informed consent statements in compliance with the guidelines of the Institutional Review Board at the University of Mary Hardin-Baylor (UMHB). All anthropometric and hemodynamic/ECG testing was conducted in the Human Performance Laboratory (HPL) and all blood processing was conducted in the Exercise Biochemistry Laboratory at UMHB.

### Baseline (T1) testing

Prior to the baseline session, subjects were instructed to record all food intakes on dietary record forms for 3 days (3-d). At the end of the 3 days, diet logs were brought back to the lab to be entered into Esha Food Processor (ESHA Research, Salem, OR) for nutritional assessment. For all testing sessions, subjects were instructed to refrain from exercise for 48 h, abstain from smoking, caffeine, tobacco as well as fast for 12-h prior to baseline testing and refrain from alcohol consumption for 24 h. Subjects were encouraged to consume plenty of water the day prior to each testing session and to consume water upon waking prior to reporting to the lab.

The day of baseline testing (T1), subjects reported to the HPL during their scheduled time (between the hours of 0500–0800) to be weighed via TANITA electronic scale (Model TBF-310, TANITA, Arlington Heights, IL) and height measured using a SECA 242 instrument (242, SECA, Hanover, MD). Resting heart rate (RHR) and blood pressure (SBP & DBP) were obtained in a rested and seated position via OMRON Digital Blood Pressure Monitor (Model HEM-907XL, OMRON Healthcare, Inc. IL U.S.A.). HR, recorded as beats per minute, SBP and DBP, recorded as mmHg, were measured at the completion of the ECG. Body composition was determined using bioelectrical impedance analysis via InBody (Model 770, InBody Co., Ltd, Cerritos, CA). For InBody measurements, test-retest reliability in our lab are as follows: Fat Mass: ICC = 0.99; Lean Mass: ICC = 0.99; percent body fat: ICC = 0.998. All InBody tests were conducted by the same technician and strict manufacturer guidelines for calibration and testing procedures were followed.

### Electocardiogram (ECG)

Once body composition was completed, subjects performed an electrocardiogram (ECG). Leads were placed in standard clinical fashion to produce a 12 lead ECG (I-III, V1-V6, aVR, aVL, aVF). Subjects remained in a supine position for 5 min. Cardiac rhythm was monitored through a Quinton Eclipse Premier Electrocardiograph (Cardiac Science Corporation, Bothell, WA). Data was printed from the 12-lead ECG machine and RR interval, RP interval, QRS duration, and QT interval were recorded.

### Blood collection protocol and questionnaires

Blood was collected from an antecubital vein with the subject in a supine position. To negate diurnal variation in hormonal status sampling was taken at the same time for each of the testing days. Upon extracting the blood into two 7.5 ml tiger top Vacutainer®, the sample was inverted gently (5–6 times) and was sent to the blood analysis room to allow time to clot and was centrifuged for 15 min within 30–60 min at room temperature to yield serum samples for complete metabolic and lipid panel measures. A 4 mL purple top Vacutainer® was drawn from the forearm vein and was mixed gently via inversion and then refrigerated for CBC measurements. All samples were then refrigerated and sent to Quest (Quest Diagnostics Inc., Irvine, TX) for analysis. All blood collection and processing protocols were in line with the recommendations from Quest diagnostics to ensure optimal samples for analysis.

After blood collection subjects were given the supplement based on their randomly assigned group and told to ingest the supplement with 8 fluid ounces of water and remained in the HPL for 30 min to ensure no allergic reaction occurred. Immediately after ingestion of the supplement, subjects were given a symptoms survey, Profile of Mood States (POMS), the Visual Analog Scale (VAS) (including: energy, focus, concentration, sleep, vigor, and motivation to exercise), and YALE Physical Activity Survey (YALE) to establish baseline measures of these dependent variables. The VAS was structured as a 15-cm scale that was labeled as “lowest possible” and “highest possible” for each VAS variable as used in previous research [10, 11]. The YALE survey has been used in various settings as a measure of physical activity [12, 13].

### Supplementation protocol

Subjects were matched according to T1 body mass and gender, then in a randomized, double-blind manner were assigned to consume a low dose of 200 mg of TeaCrine® (TC-LD) (n = 19, 23.5 ± 6 years, 175.6 ± 13.3 cm, 78 ± 18.4 kg, 23.4 ± 10 %BF), a higher dose of 300 mg of TeaCrine® (TC-HD) (n = 21, 21.38 ± 2.3 years, 172.7 ± 12 cm, 77 ± 17.3 kg, 24 ± 10.2 %BF) or 300 mg of maltodextrin (PLA) (n = 20, 22.8 ± 4.7 years, 174 ± 12.1 cm, 77 ± 17.2 kg, 22.3 ± 9.9 %BF) once per day, after breakfast but before lunch, with 8 oz of water for the 8-week study protocol. Dosing was selected based on previous research that has been presented at the ISSN Annual Meeting [2]. The terms high and low dose are relative to what was utilized in this study and future research is needed to truly establish what determines a true “high” and “low” dose of TeaCrine®. Compliance of pill ingestion was monitored by research assistants every 2 weeks in the HPL when subjects would return to get a new 2 week supply of pills for their respective treatment group.

### Follow-up testing and dietary intake

Throughout the 8-week protocol, subjects returned to the HPL for follow-up testing at week 4 (T2) and week 8 (T3) and repeated identical testing procedures as discussed during T1 testing. Note that prior to T1 testing subjects were instructed to record all food intakes on 3-d dietary record forms. Subsequently, subjects were told to maintain their diets as closely as possible to the previously 3-d recorded intakes (on T1). Dietary intakes for calories and macronutrient profiles are displayed in Table 1.

**Table 1:** Macronutrient intakes

Variable	T1	T2	T3	Time*group	Time*group*gender
kcal/d					
PLA	2054 ± 700	2132 ± 822	2128 ± 778	Time *p* = 0.11	G*T*Ge *p* = 0.17
LD	2469 ± 727	2450 ± 957	2155 ± 563	G*T *p* = 0.39	
HD	2478 ± 1252	2395 ± 1139	2031 ± 763		
Fat g/d					
PLA	85 ± 30	83 ± 32	92 ± 50	Time *p* = 0.89	G*T*Ge *p* = 0.41
LD	101 ± 47	105 ± 50	104 ± 30	G*T *p* = 0.42	
HD	100 ± 60	94 ± 55^a^	83 ± 35^a^		
Protein g/d					
PLA	106 ± 32	94 ± 28	96 ± 36	**Time** ***p*** **= 0.04**	G*T*Ge *p* = 0.40
LD	108 ± 62	101 ± 46	90 ± 34	G*T *p* = 0.48	
HD	110 ± 69	108 ± 57	83 ± 48		
Carb g/d					
PLA	223 ± 109	320 ± 297	234 ± 91	Time *p* = 0.23	G*T*Ge *p* = 0.37
LD	284 ± 102	284 ± 119	266 ± 89	G*T *p* = 0.52	
HD	282 ± 127	280 ± 148	235 ± 102		

Abbreviations: *PLA* Placebo, *LD* Theacrine Low Dose, *HD* Theacrine High Dose, *G* Group, *T* Time, *Ge* Gender

Superscript letters, different letters are significantly different at a given time point (*p* < 0.05). Items in bold indicate a significant effect (*p* < 0.05) was observed

### Statistics

Statistical analyses was performed using SPSS v22.0 (Chicago, IL, USA). An alpha level (α) of *p* ≤ 0.05 was used to determine significance within or between groups.

Mixed-factorial ANOVA’s with repeated measures [group (PLA × TC-LD × TC-HD) × gender (M × F) × time (T1, T2, T3)] were used for all dependent variables. If a significant [(time), (time*group), (time*group*gender)] interaction was observed, additional post hoc analyses were performed as follows: 1) for significant time effects, within-group repeated measures ANOVAs with Bonferroni’s corrections were performed, 2) for significant time*group interactions, within-group repeated measures ANOVAs with Bonferroni’s corrections, and one-way ANOVAs at each time point with Tukey post hoc analyses were performed, and 3) for significant time*group*gender interactions, within-group repeated measures ANOVAs with Bonferroni’s corrections were performed for each gender, and one-way ANOVAs with Tukey post hoc analyses were performed for each gender. Unless otherwise noted, data are presented as mean ± SD.

## Results

### Effects of TeaCrine® on macronutrient intakes

The effects of each treatment on macronutrient intakes are listed in Table 1. Analyses for protein/d revealed a main effect for time (*p* = 0.04), but no time*group interaction (*p* = 0.48). Split-plot analyses for protein/d regarding time*group*gender interactions revealed no interaction (*p* = 0.40). A Tukey’s post hoc for the main time effect revealed a significant difference between T1 and T3 (*p* = 0.042); however, T1 and T3 comparisons within each group revealed no significant differences (*p* > 0.05). Analyses for kcal/d, carb/d and fat/d revealed no main effect for time, time*group interaction or time*group*gender interaction (data in Table 1).

### Effects of TeaCrine® on body composition

**Fig. 1:**
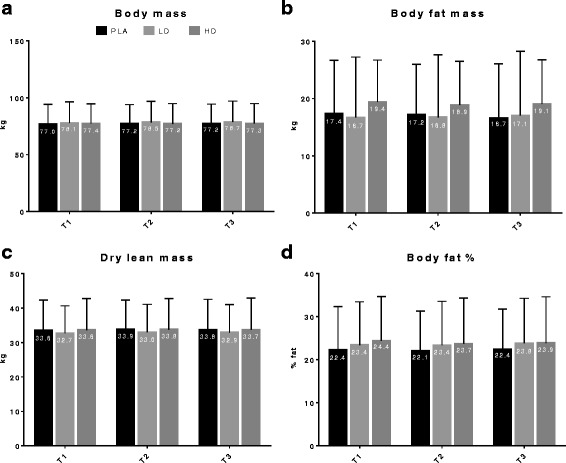
Between-treatment effects on body composition variables are illustrated in graph (**a**) Body mass, (**b**) Body fat mass, (**c**) Dry lean mass, and (**d**) Body fat %. Abbreviations: PLA, Placebo; LD, Theacrine Low Dose; HD, Theacrine High Dose. There were no main effects of time, group*time interactions or group*time*gender interactions for select body composition variables

The effects of each treatment on body composition are listed in Fig. 1. Analyses for lean mass revealed a main effect for time (*p* = 0.023), but no time*group interaction (*p* = 0.99). Split-plot analyses for lean mass regarding time*group*gender interactions revealed no interaction (*p* = 0.19). Additional post hoc analyses revealed a main effect between T1 and T2 (*p* = 0.037); however, T1 and T2 comparisons within each group revealed no significant differences (*p* > 0.05). Analyses for body weight, fat mass and body fat percentage revealed no main effects for time, time*group interactions or time*group*gender interactions (Fig. 1).

### Effects of TeaCrine® on clinical safety markers

#### Blood pressure, heart rate, and ECG variables

The effects of each treatment on cardiovascular safety markers are listed in Tables 2 and 3. Analyses for heart rate revealed a main effect for time (*p* = 0.032), but no main effect for time*group interaction (*p* = 0.35). Split-plot analyses for heart rate regarding time*group*gender interactions revealed no interaction (*p* = 0.85). Additional post hoc analyses revealed a main effect between T1 and T3 (*p* = 0.049); however, T1 and T3 comparisons within each group revealed no significant differences (*p* > 0.05). Analyses for SBP, DBP and Vent Rate revealed no main effects for time, time*group interactions or time*group*gender interactions (Table 2).

**Table 2:** Cardiovascular safety markers, part I

Variable	T1	T2	T3	Time*group	Time*group*gender
SBP (mm Hg)					
PLA	117 ± 15	116 ± 12	115 ± 13	Time *p* = 0.45	G*T*Ge *p* = 0.73
LD	116 ± 9	115 ± 8	116 ± 6	G*T *p* = 0.12	
HD	118 ± 15	124 ± 12	122 ± 13		
DBP (mm Hg)					
PLA	69 ± 9	68 ± 9	68 ± 9	Time *p* = 0.58	G*T*Ge *p* = 0.89
LD	70 ± 7	67 ± 8	71 ± 8	G*T *p* = 0.53	
HD	70 ± 8	68 ± 8	73 ± 8		
Heart rate (beats/min)					
PLA	60 ± 10	64 ± 8	63 ± 10	**Time** ***p*** **= 0.032**	G*T*Ge *p* = 0.85
LD	60 ± 15	59 ± 10^a^	63 ± 12^a^	G*T *p* = 0.35	
HD	59 ± 12	63 ± 10	64 ± 13		
Vent Rate (breath/min)					
PLA	60 ± 9	61 ± 10	62 ± 8	Time *p* = 0.43	G*T*Ge *p* = 0.67
LD	57 ± 13	59 ± 11	60 ± 13	G*T *p* = 0.10	
HD	63 ± 12	59 ± 11	61 ± 11		

Abbreviations: *PLA* Placebo, *LD* Theacrine Low Dose, *HD* Theacrine High Dose, *G* Group, *T* Time, *Ge* Gender

Superscript letters, different letters are significantly different at a given time point (*p* < 0.05). Items in bold indicate a significant effect (*p* < 0.05) was observed

**Table 3:** Cardiovascular safety markers, part II

Variable	T1	T2	T3	Time*group	Time*group*gender
RR Interval (ms)					
PLA	1011 ± 148	991 ± 147	976 ± 113	Time *p* = 0.23	G*T*Ge *p* = 0.68
LD	1095 ± 240	1041 ± 185	1035 ± 212	G*T *p* = 0.55	
HD	987 ± 213	1043 ± 221	1007 ± 198		
PR Interval (ms)					
PLA	155 ± 25	155 ± 18	156 ± 20	Time *p* = 0.49	G*T*Ge *p* = 0.42
LD	153 ± 18	153 ± 20	154 ± 17	G*T *p* = 0.33	
HD	150 ± 24	144 ± 24	143 ± 19		
QRS Duration (ms)					
PLA	95 ± 11	91 ± 22	96 ± 12	Time *p* = 0.32	G*T*Ge *p* = 0.25
LD	93 ± 12	93 ± 11	94 ± 11	G*T *p* = 0.41	
HD	92 ± 13	92 ± 12	92 ± 12		
QT Interval (ms)					
PLA	428 ± 27	424 ± 31	424 ± 25	Time *p* = 0.33	G*T*Ge *p* = 0.38
LD	427 ± 40	424 ± 36	419 ± 38	G*T *p* = 0.36	
HD	409 ± 26	614 ± 904	418 ± 38		
QTc Interval (ms)					
PLA	429 ± 17	426 ± 19	427 ± 22	Time *p* = 0.89	G*T*Ge *p* = 0.35
LD	421 ± 24	422 ± 22	419 ± 23	G*T *p* = 0.33	
HD	414 ± 19	417 ± 20	419 ± 27		
QT Dispersion (ms)					
PLA	38 ± 25	33 ± 18	36 ± 42	Time *p* = 0.78	G*T*Ge *p* = 0.11
LD	35 ± 21	51 ± 90	38 ± 25	G*T *p* = 0.51	
HD	41 ± 27	32 ± 21	32 ± 27		
P Axis (degrees)					
PLA	54 ± 31	65 ± 12	59 ± 25	Time *p* = 0.66	G*T*Ge *p* = 0.88
LD	52 ± 37	53 ± 37	53 ± 35	G*T ***p*** **= 0.033**	
HD	61 ± 15	54 ± 19	59 ± 17		
R Axis (degrees)					
PLA	73 ± 32	78 ± 26	76 ± 33	Time *p* = 0.81	G*T*Ge *p* = 0.13
LD	74 ± 25	67 ± 33	72 ± 26	G*T *p* = 0.38	
HD	85 ± 9	84 ± 9	84 ± 11		
T Axis (degrees)					
PLA	54 ± 20	415 ± 1587	59 ± 14	Time *p* = 0.15	G*T*Ge *p* = 0.34
LD	61 ± 17	438 ± 1653	60 ± 17	G*T *p* = 0.90	
HD	200 ± 634	312 ± 1160	60 ± 17		

Abbreviations: *PLA* Placebo, *LD* Theacrine Low Dose, *HD* Theacrine High Dose, *G* Group, *T* Time, *Ge* Gender

Items in bold indicate a significant effect (*p* < 0.05) was observed

Analyses for p axis revealed no main effect for time (*p* = 0.66), but revealed a main effect for time*group interaction (*p* = 0.033). Split-plot analyses for p axis regarding time*group*gender interactions revealed no interaction (*p* = 0.88). However, additional post hoc analyses revealed no significant differences within groups from T1 through T3. Moreover, one-way ANOVAs at each time point revealed no differences between each treatment. Analyses for ECG RR Interval, ECG PR Interval, ECG QRS Duration, ECG QT Interval, ECG QTc Interval, ECG QT Dispersion, ECG R Axis and ECG T Axis revealed no main effects for time, time*group interactions or time*group*gender interactions (Table 3).

#### Clinical blood safety markers

The effects of each treatment on clinical serum and whole blood safety markers are listed in Tables 4, 5, 6 and 7.

**Table 4:** Serum clinical safety markers, part I

Variable	T1	T2	T3	Time*group	Time*group*gender
Cholesterol (mg/dL)					
PLA	155 ± 37	154 ± 34	161 ± 32	Time *p* = 0.07	G*T*Ge *p* = 0.38
LD	151 ± 28	150 ± 26	160 ± 28	G*T ***p*** **= 0.001**	
HD	**174 ± 33**	**162 ± 33** ^**†**^	**160 ± 32** ^**†**^		
HDL (mg/dL)					
PLA	49 ± 10	51 ± 12	55 ± 12	Time *p* = 0.08	G*T*Ge *p* = 0.99
LD	47 ± 11	50 ± 12	51 ± 12	G*T *p* = 0.16	
HD	53 ± 16	49 ± 15	52 ± 16		
LDL (mg/dL)					
PLA	89.4 ± 28.9	83.4 ± 28.9	87.7 ± 23.9	**Time** ***p*** **= 0.001**	G*T*Ge *p* = 0.38
LD	85.0 ± 19.8	78.9 ± 17.8	86.9 ± 22.2	G*T ***p*** **= 0.024**	
HD	**103.0 ± 22.1**	**89.3 ± 20.5** ^**†**^	**89.2 ± 19.9** ^**†**^		
Cholesterol/HDL					
PLA	3.3 ± 0.9	3.1 ± 0.9	3.0 ± 0.8	Time *p* = 0.19	G*T*Ge *p* = 0.48
LD	3.2 ± 0.8	3.0 ± 0.8	3.2 ± 0.9	G*T *p* = 0.77	
HD	35 ± 0.9	3.3 ± 0.9	3.3 ± 0.9		
Triglycerides (mg/dL)					
PLA	67 ± 25	77 ± 31	73 ± 31	Time *p* = 0.21	G*T*Ge *p* = 0.73
LD	80 ± 45	93 ± 50	97 ± 50	G*T *p* = 0.63	
HD	87 ± 39	92 ± 40	83 ± 36		
Glucose (mg/dL)					
PLA	86 ± 8	86 ± 8	86 ± 6	Time *p* = 0.14	G*T*Ge *p* = 0.44
LD	87 ± 7	86 ± 8	90 ± 8	G*T *p* = 0.55	
HD	84 ± 9	86 ± 9	86 ± 7		

Abbreviations: *PLA* Placebo, *LD* Theacrine Low Dose, *HD* Theacrine High Dose, *G* Group, *T* Time, *Ge* Gender

Symbols: ^†^, different from corresponding T1 value (*p* < 0.05). Items in bold indicate a significant effect (*p* < 0.05) was observed

**Table 5:** Serum clinical safety markers, part II

Variable	T1	T2	T3	Time*group	Time*group*gender
Urea Nitrogen (mg/dL)					
PLA	16 ± 4	15 ± 4	16 ± 5	Time *p* = 0.89	G*T*Ge *p* = 0.39
LD	14 ± 3	14 ± 3	15 ± 3	G*T *p* = 0.34	
HD	15 ± 4	13 ± 4	14 ± 3		
Creatinine (mg/dL)					
PLA	1.18 ± 0.2	0.96 ± 0.2	0.97 ± 0.2	Time *p* = 0.33	G*T*Ge ***p*** **= 0.046**
LD	0.97 ± 0.1	1.02 ± 0.2	**1.03 ± 0.2** ^**‡**^	G*T *p* = 0.06	
HD	0.99 ± 0.2	1.01 ± 0.2	1.05 ± 0.2		
Sodium (mmoL)					
PLA	140.65 ± 3	139.85 ± 2	140.95 ± 2	**Time** ***p*** **= 0.007**	G*T*Ge *p* = 0.84
LD	141.22 ± 2	140.17 ± 2	140.95 ± 3	G*T *p* = 0.67	
HD	141.19 ± 3	**139.95 ± 2** ^**†**^	140.24 ± 2		
Potassium (mmoL)					
PLA	4.9 ± 0.4	4.7 ± 0.6	4.9 ± 0.5	Time *p* = 0.22	G*T*Ge *p* = 0.99
LD	5.0 ± 0.6	4.8 ± 0.4	4.9 ± 0.5	G*T *p* = 0.97	
HD	4.9 ± 0.4	4.8 ± 0.4	4.9 ± 0.4		
Chloride (mmoL)					
PLA	105.60 ± 2	**104.45 ± 2** ^**†**^	105.00 ± 2	**Time** ***p*** **= 0.006**	G*T*Ge *p* = 0.60
LD	105.89 ± 2	105.33 ± 2	104.84 ± 2	G*T *p* = 0.78	
HD	105.24 ± 2	**104.33 ± 2** ^**†**^	104.57 ± 2		
Calcium (mg/dL)					
PLA	9.7 ± 0.5	9.5 ± 0.4	9.6 ± 0.4	Time *p* = 0.37	G*T*Ge *p* = 0.74
LD	9.6 ± 0.3	9.6 ± 0.4	9.6 ± 0.3	G*T *p* = 0.20	
HD	9.8 ± 0.3	9.7 ± 0.4	9.7 ± 0.3		
Carbon Dioxide (mmoL)					
PLA	24 ± 2	25 ± 2	24 ± 2	Time *p* = 0.65	G*T*Ge *p* = 0.95
LD	26 ± 2	26 ± 2	25 ± 2	G*T *p* = 0.85	
HD	25 ± 2	25 ± 2	25 ± 2		

Abbreviations: *PLA* Placebo, *LD* Theacrine Low Dose, *HD* Theacrine High Dose, *G* Group, *T* Time, *Ge* Gender

Symbols: ^‡^, increase from corresponding T1 value within the male TC-LD group (*p* < 0.05); ^†^, different from corresponding T1 value (*p* < 0.05). Items in bold indicate a significant effect (*p* < 0.05) was observed

**Table 6:** Serum clinical safety markers, part III

Variable	T1	T2	T3	Time*group	Time*group*gender
Serum protein (g/dL)					
PLA	7.0 ± 0.4	6.8 ± 0.4	7.0 ± 0.4	**Time** ***p*** **= 0.011**	G*T*Ge *p* = 0.97
LD	7.0 ± 0.5	6.8 ± 0.4	7.0 ± 0.4	G*T ***p*** **= 0.006**	
HD	7.2 ± 0.4	7.0 ± 0.5	**6.9 ± 0.3** ^**†**^		
Albumin (g/dL)					
PLA	4.6 ± 0.4	4.4 ± 0.4	4.5 ± 0.4	**Time** ***p*** **= 0.023**	G*T*Ge *p* = 0.67
LD	4.5 ± 0.3	4.5 ± 0.3	4.6 ± 0.3	G*T *p* = 0.17	
HD	4.5 ± 0.3	4.5 ± 0.3	4.4 ± 0.2		
Globulin (g/dL)					
PLA	2.5 ± 0.3	2.4 ± 0.3	2.5 ± 0.2	Time *p* = 0.15	G*T*Ge *p* = 0.85
LD	2.4 ± 0.4	2.3 ± 0.3	2.5 ± 0.3	G*T *p* = 0.10	
HD	2.6 ± 0.4	2.5 ± 0.4	2.4 ± 0.4		
Albumin/Globulin					
PLA	1.9 ± 0.3	1.9 ± 0.3	1.9 ± 0.3	Time *p* = 0.76	G*T*Ge *p* = 0.72
LD	1.9 ± 0.3	1.9 ± 0.3	1.9 ± 0.2	G*T *p* = 0.66	
HD	1.8 ± 0.4	1.8 ± 0.4	1.8 ± 0.3		
Bilirubin (mg/dL)					
PLA	0.7 ± 0.3	0.7 ± 0.4	0.8 ± 0.5	Time *p* = 0.84	G*T*Ge *p* = 0.40
LD	0.7 ± 0.3	0.7 ± 0.3	0.6 ± 0.3	G*T *p* = 0.44	
HD	0.7 ± 0.3	0.7 ± 0.3	0.6 ± 0.3		
ALP (IU/L)					
PLA	58 ± 17	57 ± 15	58 ± 15	Time *p* = 0.47	G*T*Ge *p* = 0.36
LD	53 ± 11	55 ± 12	57 ± 12	G*T *p* = 0.25	
HD	65 ± 13	60 ± 15	62 ± 13		
AST (IU/L)					
PLA	21 ± 6	19 ± 5	20 ± 4	Time *p* = 0.12	G*T*Ge *p* = 0.66
LD	19 ± 5	18 ± 4	20 ± 7	G*T *p* = 0.81	
HD	20 ± 6	18 ± 6	20 ± 7		
ALT (IU/L)					
PLA	17 ± 7	17 ± 6	17 ± 6	Time *p* = 0.34	G*T*Ge *p* = 0.45
LD	17 ± 7	17 ± 8	23 ± 24	G*T *p* = 0.19	
HD	19 ± 8	17 ± 12	17 ± 6		

Abbreviations: *PLA* Placebo, *LD* Theacrine Low Dose, *HD* Theacrine High Dose, *G* Group, *T* Time, *Ge* Gender

Symbols: ^†^, different from corresponding T1 value (*p* < 0.05). Items in bold indicate a significant effect (*p* < 0.05) was observed

**Table 7:** Clinical whole blood safety markers, part I

Variable	T1	T2	T3	Time*group	Time*group*gender
White blood cells (10^3^ cells/μL)					
PLA	6.0 ± 1.8	5.9 ± 1.7	6.2 ± 1.6	Time *p* = 0.52	G*T*Ge *p* = 0.18
LD	6.5 ± 1.5	6.3 ± 1.5	6.4 ± 1.5	G*T *p* = 0.52	
HD	6.9 ± 1.5	6.5 ± 1.2	6.4 ± 1.8		
RBC (10^6^ cells/μl)					
PLA	4.4 ± 0.4	4.4 ± 0.5	4.5 ± 0.4	Time *p* = 0.12	G*T*Ge *p* = 0.10
LD	4.8 ± 0.4	4.7 ± 0.4	4.8 ± 0.4	G*T *p* = 0.36	
HD	4.8 ± 0.4	4.8 ± 0.5	4.8 ± 0.5		
Hemoglobin (g/dL)					
PLA	13.8 ± 1.0	13.7 ± 1.5	**13.9 ± 1.3** ^**‡**^	Time *p* = 0.56	G*T*Ge ***p*** **= 0.006**
LD	14.4 ± 1.1	14.3 ± 1.1	14.5 ± 1.1	G*T *p* = 0.78	
HD	14.2 ± 1.4	14.1 ± 1.3	14.1 ± 1.4		
Hematocrit (%)					
PLA	41 ± 3	41 ± 5	**42 ± 3** ^**‡**^	Time *p* = 0.23	G*T*Ge ***p*** **= 0.049**
LD	43 ± 4	43 ± 3	44 ± 3	G*T *p* = 0.85	
HD	43 ± 4	43 ± 4	43 ± 4		
Platelet Count (10^3^ particles/μL)					
PLA	160 ± 46	**191 ± 50** ^**†**^	204 ± 89	**Time** ***p*** **= 0.005**	G*T*Ge *p* = 0.16
LD	148 ± 61	176 ± 35	212 ± 71	G*T *p* = 0.77	
HD	191 ± 40	216 ± 51	227 ± 41		
Neutrophils (10^3^ cells/μL)					
PLA	3123 ± 843	3441 ± 1440	3679 ± 1443	Time *p* = 0.67	G*T*Ge *p* = 0.12
LD	3423 ± 920	3196 ± 862	3444 ± 881	G*T *p* = 0.28	
HD	3631 ± 1417	3570 ± 921	3452 ± 1423		
Lymphocytes (10^3^ cells/μL)					
PLA	1881 ± 543	1908 ± 479	1928 ± 492	Time *p* = 0.20	G*T*Ge *p* = 0.50
LD	2393 ± 698	2325 ± 738	2232 ± 681	G*T *p* = 0.13	
HD	2624 ± 664	2200 ± 425	2384 ± 761		
Monocytes (10^3^ cells/μL)					
PLA	426 ± 123	414 ± 150	412 ± 131	Time *p* = 0.97	G*T*Ge *p* = 0.07
LD	467 ± 149	498 ± 131	456 ± 156	G*T *p* = 0.65	
HD	424 ± 184	399 ± 106	427 ± 123		
Eosinophils (10^3^ cells/μL)					
PLA	157 ± 109	**187 ± 125** ^**†**^	166 ± 115	Time *p* = 0.62	G*T*Ge ***p*** **= 0.002**
LD	187 ± 108	187 ± 102	189 ± 112	G*T ***p*** **= 0.041**	
HD	225 ± 130	164 ± 87	181 ± 83		
Basophils (10^3^ cells/μL)					
PLA	27 ± 12	24 ± 11	25 ± 12	Time *p* = 0.07	G*T*Ge *p* = 0.28
LD	30 ± 14	36 ± 19	25 ± 11	G*T *p* = 0.21	
HD	32 ± 18	43 ± 23	33 ± 20		

Abbreviations: *PLA* Placebo, *LD* Theacrine Low Dose, *HD* Theacrine High Dose, *G* Group, *T* Time, *Ge* Gender

Symbols: ^‡^, increase from corresponding T2 value within the female PLA group (*p* < 0.05); ^†^, different from corresponding T1 value (*p* < 0.05). Items in bold indicate a significant effect (*p* < 0.05) was observed

Analyses for total cholesterol revealed no main effect for time (*p* = 0.07), but revealed a main effect for time*group interaction (*p* = 0.001). Split-plot analyses for total cholesterol regarding time*group*gender interactions revealed no interaction (*p* = 0.86). Lower cholesterol levels (*p* ≤ 0.05) were revealed within the TC-HD group at T2 and T3 compared to T1. Analyses for LDL revealed a main effect for time (*p* = 0.001), and time*group interaction (*p* = 0.024). Split-plot analyses for LDL regarding time*group*gender interactions revealed no interaction (*p* = 0.38). Significant differences (*p* ≤ 0.05) were revealed within the TC-HD group at T2 and T3 compared to T1. Analyses for HDL cholesterol, triglycerides, CHOL/HDL ratio, and glucose levels revealed no main effects for time, time*group interactions or time*group*gender interactions (Table 4).

Analyses for serum creatinine revealed no main effect for time (*p* = 0.33), or time*group interaction (*p* = 0.06), although there was a time*group*gender interaction (*p* = 0.039). Additional post hoc analyses revealed creatinine to increase within the male TC-LD subjects from T1 to T3 (means: 1.05 → 1.15, *p* < 0.05), although levels remained within clinical reference ranges. Analyses for serum sodium revealed a main effect for time (*p* = 0.007), but no time*group interaction (*p* = 0.67) or time*group*gender interaction (*p* = 0.84). Additional post hoc analyses revealed sodium decreased (*p* ≤ 0.05) at T2 compared to T1 in the TC-HD group. Analyses for serum chloride revealed a main effect for time (*p* = 0.006), but no time*group interaction (*p* = 0.97) or time*group*gender interaction (*p* = 0.60). However, post-hoc analyses revealed no within- or between-group changes in serum chloride (*p* > 0.05). Analyses for serum urea nitrogen, potassium, calcium and carbon dioxide levels revealed no main effects for time, time*group interactions or time*group*gender interactions (Table 5).

Analyses for serum total protein revealed a main effect for time (*p* = 0.011), and time*group interaction (*p* = 0.006), although no time*group*gender interaction (*p* = 0.97). Additional post hoc analyses revealed total protein to decrease in TC-HD from T1 to T3 (*p* < 0.05), although levels remained within clinical reference ranges. Analyses for Albumin revealed a main effect for time (*p* = 0.023), but no time*group interaction (*p* = 0.17) or time*group*gender interaction (*p* = 0.67). However, additional post hoc analyses revealed no differences within- or between groups. Analyses for serum globulin, bilirubin, ALP, AST, or ALT levels revealed no main effects for time, time*group interactions or time*group*gender interactions (Table 6).

Analyses for hemoglobin revealed no main effect for time (*p* = 0.56) or time*group interaction (*p* = 0.77), but a time*group*gender interaction (*p* = 0.006). Additional post hoc analyses revealed hemoglobin at T3 to be greater than T2 for PLA females (*p* < 0.05). Analyses for hematocrit revealed no main effect for time (*p* = 0.23) or time*group interaction (*p* = 0.85), but a time*group*gender interaction (*p* = 0.049). Additional post hoc analyses revealed hematocrit at T3 to be greater than T2 for PLA females (*p* < 0.05). Analyses for platelet count revealed a main effect for time (*p* = 0.005), but no time*group interaction (*p* = 0.77) or time*group*gender interaction (*p* = 0.16). Additional post hoc analyses revealed platelets to increase in the PLA group from T1 to T2 (*p* < 0.05).

Analyses for eosinophils revealed no main effect for time (*p* = 0.62), but revealed a time*group interaction (*p* = 0.041) and time*group*gender interaction (*p* = 0.002). Additional post hoc analyses revealed eosinophils to decrease in the PLA group from T1 to T2 (*p* < 0.05), but additional gender post hocs revealed no between or within-group differences. Analyses for white blood cells, red blood cells, neutrophils, lymphocytes, monocytes, and basophils revealed no main effects for time, time*group interactions or time*group*gender interactions (Table 7).

### Effects of Teacrine® on mood and concentration profiles

Analyses for POMS Vigor revealed a main effect for time (*p* = 0.021), a time*group interaction (*p* = 0.006), and a time*group*gender interaction (*p* = 0.044). Additional post hoc analyses revealed a significant decrease in POMS vigor in the TC-LD group from T1 to T2 (*p* < 0.05), but was not different between T1 and T3. Moreover, this POMS vigor increased from T2 to T3 within the female TC-LD group (*p* < 0.05). Analyses for POMS fatigue revealed a main effect for time (*p* = 0.031), but no time*group interaction (*p* = 0.41) or time*group*gender interaction (*p* = 0.79). Additional post hoc analyses for POMS fatigue revealed no main effects between time points. Analyses for total POMS score revealed a main effect for time (*p* = 0.023), but no time*group interaction (*p* = 0.50) or time*group*gender interaction (*p* = 0.62). Additional post hoc analyses for total POMS score revealed no main effects between time points. Analyses for all other POMS variables revealed no main effects for time, time*group interactions or time*group*gender interactions (Table 8).

**Table 8:** Mood and concentration profiles

Variable	T1	T2	T3	Time*group	Time*group*gender
POMS Tension					
PLA	13 ± 4	13 ± 5	14 ± 4	Time *p* = 0.09	G*T*Ge *p* = 0.61
LD	13 ± 3	14 ± 5	14 ± 4	G*T *p* = 0.98	
HD	14 ± 4	15 ± 4	15 ± 4		
POMS Depression					
PLA	16 ± 3	16 ± 4	17 ± 4	Time *p* = 0.12	G*T*Ge *p* = 0.47
LD	16 ± 2	17 ± 5	17 ± 3	G*T *p* = 0.55	
HD	17 ± 2	18 ± 6	17 ± 5		
POMS Anger					
PLA	13.3 ± 2.4	13.9 ± 2.8	14.0 ± 3.7	Time *p* = 0.50	G*T*Ge *p* = 0.97
LD	13.0 ± 1.8	13.7 ± 4.2	13.5 ± 3.6	G*T *p* = 0.65	
HD	13.7 ± 2.4	13.4 ± 2.1	13.5 ± 2.9		
POMS Vigor					
PLA	22 ± 5	22 ± 6	21 ± 6	**Time** ***p*** **= 0.021**	G*T*Ge ***p*** **= 0.044**
LD	23 ± 5	**19 ± 6** ^**†**^	**23 ± 6** ^**‡**^	G*T ***p*** **= 0.006**	
HD	21 ± 5	20 ± 6	19 ± 5		
POMS Fatigue					
PLA	11 ± 3	12 ± 5	14 ± 5	**Time** ***p*** **= 0.031**	G*T*Ge *p* = 0.79
LD	11 ± 4	12 ± 5	11 ± 4	G*T *p* = 0.41	
HD	13 ± 5	14 ± 5	15 ± 6		
POMS Confusion					
PLA	12 ± 2	13 ± 3	13 ± 3	Time *p* = 0.64	G*T*Ge *p* = 0.22
LD	13 ± 2	13 ± 2	13 ± 3	G*T *p* = 0.65	
HD	13 ± 3	14 ± 4	13 ± 3		
POMS Total					
PLA	44 ± 13	45 ± 17	49 ± 16	**Time** ***p*** **= 0.023**	G*T*Ge *p* = 0.62
LD	43 ± 10	50 ± 20	45 ± 15	G*T *p* = 0.50	
HD	51 ± 15	58 ± 23	58 ± 20		

Abbreviations: *PLA* Placebo, *LD* Theacrine Low Dose, *HD* Theacrine High Dose, *G* Group, *T* Time, *Ge* Gender

Symbols: ^‡^, increase from corresponding T2 value within the male TC-LD group (*p* < 0.05); ^†^, different from corresponding T1 value (*p* < 0.05). Items in bold indicate a significant effect (*p* < 0.05) was observed

Analyses for VAS Energy revealed a main effect for time (*p* < 0.001), but no time*group interaction (*p* = 0.12) or time*group*gender interaction (*p* = 0.48). Additional post hoc analyses for VAS Energy revealed that energy decreased in PLA from time points T1 and T3, and T2 and T3 (*p* < 0.05). Furthermore, post hoc analyses for VAS Energy revealed that energy decreased in the TC-HD group from time points T1 and T3 (*p* < 0.05). Analyses for all other VAS variables revealed no main effects for time, time*group interactions or time*group*gender interactions.

Analyses for YALE 1 did not reveal a main effect for time (*p* > 0.05), but revealed a time*group interaction (*p* = 0.019) and did not reveal a time*group*gender interaction (*p* = 0.73). Additional post hoc analyses for total YALE 1 score revealed no main effects between time points. Analyses for all other YALE variables revealed no main effects for time, time*group interactions or time*group*gender interactions.

## Discussion

The primary objective of this study was to determine the safety of TeaCrine® supplementation in human subjects over an 8-week time course. As mentioned previously, there is only limited amount of research on theacrine supplementation in humans making this study the first of its kind. Our main findings of low- and high-dose TeaCrine® supplementation included the following: 1) supplementation was apparently safe and did not alter hemodynamic measures or blood measures associated with clinical safety, 2) there was no evidence of a tachyphylactic/habituation response that is typical of neuroactive agents such as caffeine and other stimulants, 3) supplementation in humans reduced LDL and total cholesterol, and 4) supplementation did not affect body composition measures. Our findings are described in greater detail below.

### TeaCrine® supplementation in humans does not affect clinical safety markers

A variety of teas have been deemed safe for consumption with minimal side effects such as *Camellia Sinensis*, *Camellia ptilophylla* and *Camellia assamica var. kucha* [5, 14, 15]. Specifically, the acute toxicity for theacrine ingestion in mice has been previously reported [4] to be an LD_50_ of 810.6 mg/kg, which would equate to roughly 4.0 g for an individual weighing 76 kg. Notwithstanding, there has been recent concern that green tea extracts (containing a variety of catechins) may be potentially harmful in some individuals. Indeed, a comprehensive review of the literature by US Pharmacoepia [16] reported that 34 cases (up to the year 2007) existed concerning the use of green tea products and their association with liver damage. In 13 cases, serum enzymes suggestive of liver damage were elevated following green tea supplementation. Herein, we report that all subjects supplementing with low and high TeaCrine® dosages presented ALT, AST and ALP levels that were within normal clinical ranges.

It should also be noted that chronic TeaCrine® supplementation did not negatively alter the standard hemodynamic variables (heart rate and blood pressure). We are in agreement with Shan-Bing Li et al. [5] who reported that theacrine had no observable effect on either BP or HR in rats. Unlike theacrine, caffeine is the major purine alkaloid in *Camellia Sienesis* and has shown to increase BP [17]. Therefore, TeaCrine® supplementation could be a safer alternative compared to caffeine when an elevation in BP is a health concern. Collectively, our safety data suggests that the doses used in the current study were safe for human consumption.

### The effects of TeaCrine® supplementation on self-perceived vigor

In regards to cognitive measures, low dose TeaCrine® supplementation slightly decreased POMS vigor from baseline to 4 weeks of supplementation, but measures returned to baseline after 8 weeks of supplementation. Interestingly, theacrine feedings in mice have been shown to reduce ambulatory activity [18], but at relatively low doses; an effect which (in rodents) could also be a result of reduced vigor. Additionally, it has been posited that theacrine, the major purine alkaloid in *Camellia assamica var. kucha*, has potent central nervous activation on adenosine receptors that differ from caffeine [18]. Compared to theacrine, caffeine has arousal and stimulant effects that are dependent on the A_2A_AR adenosine receptors [19] whereas, theacrine could potentially act as an endogenous sleep promoter on the A_1_ and A_2A_ adenosine receptors [20] at low doses, whereas higher doses may result in increased CNS excitation and locomotor drive [3]; a finding which could explain why POMS vigor scores were slightly lower in low-dose theacrine-supplemented participants. However, more research is needed to support this hypothesis, as measures in the present study did return to baseline by the week 8 data collection visit, and the rodent data on neurophysiologic effects of theacrine are mixed.

### TeaCrine® supplementation in humans reduced LDL and total cholesterol levels

It has been reported that polyphenols in tea could inhibit the absorption of dietary cholesterol [21]. Additionally, this effect has also been reported in tea catechins, particularly their gallate esters, which are able to reduce the absorption of cholesterol in the intestines causing a hypocholesterolemic effect [22]. In a study in hypercholesterolemia individuals [23] it was reported that green tea had a lowering effect on LDL and total cholesterol levels over twelve weeks. Similarly, we reported decreases in total cholesterol and LDL cholesterol in the HD group from baseline and time points week 4 and week 8, respectively, but all markers were within normal clinical reference ranges. Therefore, as with other similar studies reporting that tea extracts favorably affect blood lipid levels, we report that high-dose theacrine supplementation favorably alters serum total and LDL cholesterol levels. Importantly, TeaCrine® supplementation may be a viable ‘nutraceutical’ alternative to cholesterol-lowering drugs but more research is needed in this area.

### TeaCrine® supplementation does not affect body composition

In a few studies, caffeine supplementation has been shown to decrease body weight and fat mass [24]. Given that theacrine is synthesized from the caffeine pathway via hydration, oxidation and methylation [18, 25, 26] we theorized it may potentially have similar effects. Moreover, there is an abundance of literature on the efficacy of *Camellia sinensis* teas such as green tea [24, 27, 28], but there is no literature to our knowledge on theacrine supplementation and its effects on body composition. We report that TeaCrine® supplementation appeared to have no observable effects on measures of body composition over the duration of the intervention. Reasons for no effects observed within this study could include: 1) the dose utilized was not high enough for humans, 2) longer supplementation periods are needed for decrements in fat mass to occur, and/or 3) unlike caffeine and other thermogenic supplements, theacrine may be a ‘mild’ thermogenic supplement that does not elicit appreciable weight loss over shorter supplementation periods relative to other thermogenic aids (i.e., citrus aurantium, caffeine and/or other combinations of ingredients). In this regard, more research is needed to fully discern as to whether TeaCrine®-containing supplements are effective at managing body weight.

## Conclusions

This is one of the first studies to our knowledge that has studied the human safety, tachyphylactic response and potential habituation to prolonged theacrine (TeaCrine®) supplementation. One limitation to our study includes the relatively short duration of the supplementation protocol. Notwithstanding, we report that theacrine supplementation did not affect body composition measures, but reduced LDL and total cholesterol. The latter finding has potential implications for theacrine supplementation as a ‘nutraceutical’ modality for hypercholesterolemic individuals. Moreover, theacrine supplementation did not alter hemodynamic measures or serum measures associated with clinical safety, and lower and higher doses appear to be well-tolerated in humans over an 8 week period.
